# Comprehensive identification of diverse ribosomal RNA modifications by targeted nanopore direct RNA sequencing and JACUSA2

**DOI:** 10.1080/15476286.2023.2248752

**Published:** 2023-08-27

**Authors:** Isabel S. Naarmann-de Vries, Christiane Zorbas, Amina Lemsara, Michael Piechotta, Felix G. M. Ernst, Ludivine Wacheul, Denis L. J. Lafontaine, Christoph Dieterich

**Affiliations:** a Section of Bioinformatics and Systems Cardiology, University Hospital Heidelberg, Heidelberg, Germany; b German Center for Cardiovascular Research (DZHK), Partner Site Heidelberg/Mannheim, Heidelberg, Germany; c RNA Molecular Biology, Université libre de Bruxelles (ULB), Fonds de la Recherche Scientifique (F.R.S./FNRS), Gosselies, Belgium

**Keywords:** RNA modification, nanopore, direct RNA-seq, ribosomal RNA, RNA methylation, cytidine acetylation

## Abstract

Ribosomal RNAs are decorated by numerous post-transcriptional modifications whose exact roles in ribosome biogenesis, function, and human pathophysiology remain largely unknown. Here, we report a targeted direct rRNA sequencing approach involving a substrate selection step and demonstrate its suitability to identify differential modification sites in combination with the JACUSA2 software. We compared JACUSA2 to other tools designed for RNA modification detection and show that JACUSA2 outperforms other software with regard to detection of base modifications such as methylation, acetylation and aminocarboxypropylation. To illustrate its widespread usability, we applied our method to a collection of CRISPR-Cas9 engineered colon carcinoma cells lacking specific enzymatic activities responsible for particular rRNA modifications and systematically compared them to isogenic wild-type RNAs. Besides the numerous 2′-O methylated riboses and pseudouridylated residues, our approach was suitable to reliably identify differential base methylation and acetylation events. Importantly, our method does not require any prior knowledge of modification sites or the need to train complex models. We further report for the first time detection of human rRNA modifications by direct RNA-sequencing on Flongle flow cells, the smallest-scale nanopore flow cell available to date. The use of these smaller flow cells reduces RNA input requirements, making our workflow suitable for the analysis of samples with limited availability and clinical work.

## Introduction

Ribosomes are evolutionary-conserved sophisticated nanomachines responsible for protein production in all three kingdoms of life. They are composed of a small and a large subunit, termed 40S and 60S in eukaryotes. Mammalian ribosomes are composed of four ribosomal RNAs (rRNAs), the 5S, 5.8S, 18S, and 28S rRNAs and of 80 ribosomal proteins [1]. The biogenesis of ribosomes is a highly complex process that requires several hundreds of assembly factors [2–5], which are either proteins or small RNAs (small nucleolar RNAs, snoRNAs).

An important step of ribosome biogenesis is the modification of rRNAs [6,7]. Some modifications are conserved throughout kingdoms, others are not. In eukaryotes, the most abundant rRNA modifications are the 2′-O-methylation of ribose (sugar methylation) and the isomerization of uridine to pseudouridine (Ψ) [8]. In a recent comprehensive study conducted on human lymphoblast TK6 cells, 42 pseudouridines and 42 2′-O-ribose methylations were described on the 18S rRNA, whereas the 28S rRNA was shown to contain 61 pseudouridines and 68 2′-O-ribose methylations [8]. Besides these abundant modifications, rRNAs carry additional base modifications, including methylation, acetylation, and aminocarboxypropylation [8]. Most activities responsible for rRNA modifications have been identified to date [1], however their biological role is often only partially understood. In some cases, it is the physical presence of the modification enzyme in cells rather than that of the modification which is important [9]. 2′-O-methylation and pseudouridylation are catalysed by snoRNA-guided enzyme complexes [10] and are thought to stabilize secondary and tertiary structures of modified rRNAs [11,12]. Furthermore, some of them at least are required to promote efficient and proper translation [12–14].

The importance of correct ribosome assembly for cell homoeostasis is evidenced by an emerging class of syndromes designated as ribosomopathies. Ribosomopathies are ribosome biogenesis dysfunction diseases caused by ribosomal protein or ribosome assembly factor mutations [15]. Although all ribosomopathies affect ribosome biogenesis to some extent, they give rise to different syndromes [15]. For example, mutations in the snoRNA-associated pseudouridine synthetase *DKC1* (dyskerin) cause X-linked dyskeratosis congenita, which is associated with bone marrow failure, skin and mucosa alterations, and increased cancer susceptibility [16]. Mutations in *DKC1* also lead to defective telomerase maintenance [17], and it is understood that, in this case, both telomerase dysfunction and aberrant RNA modification contribute to the disease. The observed decrease in pseudouridine levels is accompanied by an impairment in the translation of specific cellular mRNAs, in particular those harbouring internal ribosome entry sites in their 5′ UTR [12]. Furthermore, translational fidelity is strongly reduced by *DKC1* depletion [18,19].

In the past, the analysis of mutations in ribosomal proteins and ribosome assembly factors was made possible by the use of diverse perturbation techniques. However, it was less feasible to systematically elucidate the effect of mutations in rRNA (due to the high complexity and repetitive nature of genomic rDNA loci [20–22]), or to directly assess dynamic changes in distinct rRNA modifications in clinical or biological samples for which the amount of material is limited. Mass spectrometry- and HPLC-based methods usually require a high amount of input material (µg range) to allow for the accurate detection of ribosomal modifications [8]. Other deep-sequencing-based methods such as RiboMethSeq [23] or HydraPsiSeq [24] are restricted to specific modification types: 2′-O-ribose methylation or pseudouridine detection, respectively. Consequently, the relevance of mutations or aberrant modifications in rRNAs is not well understood.

With the advent of long-read sequencing techniques, especially the direct RNA sequencing method (direct RNA-seq) introduced by Oxford Nanopore Technologies (ONT), it has become possible to directly sequence full-length RNA molecules [25–27]. Using direct RNA-seq, RNA modifications can now also be analysed directly as exemplified recently for m^6^A [25,26,28–31] and pseudouridine [31,32]. Nanopore direct RNA-seq was previously used to analyse rRNA sequences derived from bacteria [29,30], yeast [30,32,33], and human cells [30,31]. An increasing number of computational tools are becoming available to call RNA modifications in direct RNA-seq data [30–36] with different underlying concepts. Each of these methods displays specific advantages and inherent limitations. For example, nanoRMS uses changes in the electric current intensity and trace profiles to analyse pseudouridine modifications [32]. Furthermore, the software can be adapted to identify differential 2′-O-ribose methylation sites; however, it is not intended for detection of the heterogeneous class of other base modifications. Another recent work based on signalAlign profiles known rRNA modifications at the single read level, but requires extensive prior knowledge based on a previously reported modification map [33]. Thus, this method is not suitable to identify novel modifications. We have recently introduced the JACUSA2 algorithm for the analysis of RNA modifications [31], which uses basecalling errors (Mismatch, Deletion, Insertion) in pairwise comparisons (call-2 mode), and handles replicate samples. Previously, we have demonstrated the feasibility of JACUSA2 in the analysis of m^6^A modifications on mRNA and the detection of modified uridine residues (mainly pseudouridine) on rRNA [31].

In this work, we show that JACUSA2 affords the *de novo* identification of differential modification sites with no need for training data or prior knowledge of the position and chemical nature of the modification. First, we established nanopore targeted direct RNA-seq of human 18S rRNA with a specific adapter (rather than by performing bulk polyadenylation and non-discriminative sequencing of total RNA). In a benchmark of JACUSA2 against other published tools, we find that JACUSA2 Mis is especially useful to identify base modifications as methylation and acetylation. Moreover, JACUSA2 is faster and computationally less demanding than most other tools. To validate the detection of these RNA modifications, we employed a collection of CRISPR-Cas9-engineered human colon carcinoma cells (HCT116) lacking specific modifications on the 18S rRNA. To detect modification signatures in nanopore direct rRNA-seq data, each analysed modification was systematically compared in mutant (MUT)/knock out (KO) and isogenic wild type (WT) control cells. Using this approach, we validated, in addition to the abundant 2′-O-ribose methylated and pseudouridylated residues, several 18S rRNA base modifications (including the METTL5-catalysed m^6^A_1832_ [37], the DIMT1L-catalysed m${\;}_{2}^{6}A$_1850_ m${\;}_{2}^{6}A$_1851_ [9,38,39], the WBSCR22-catalysed m^7^G_1639_ [9,40,41], and the NAT10/SNORD13-catalysed ac^4^C_1842_). Employing JACUSA2 call-2, we show that all analysed rRNA modifications are detectable as differential sites in nanopore direct rRNA-seq data. Importantly, we provide experimental and computational evidence that our approach can estimate relative levels of modification based on calibration curves. Lastly, to expand the repertoire of biological samples that are accessible to nanopore direct rRNA-seq, the targeted rRNA-seq method was transferred to the small-scale Flongle flow cells. We demonstrate that downscaled nanopore direct rRNA-seq on these small devices equipped with only 126 pores allows for detection of rRNA modifications as efficiently as with MinION flow cell sequencing.

In summary, we show that nanopore direct rRNA-seq in combination with JACUSA2 is a fast and simple approach to identify changes in rRNA modification pattern, irrespective of the chemical nature of the analysed modifications or prior knowledge on the modification sites. Furthermore, our miniature Flongle-based sequencing approach makes the method amenable to precious low-input samples of biological and clinical relevance.

## Materials and methods

### Generation of HCT116 mutant cell lines

All human cell lines were generated in p53-positive diploid HCT116 cells (ATCC, #CCL-247) by genome editing. The recipient cell line was diagnosed by ATCC by short tandem repeat (STR) analysis prior to use. The HCT116 METTL5^−/−^ cell line has been described previously [37]. Here, the exon encoding the catalytic domain of the protein was precisely excised from the genome on both alleles by CRISPR-Cas9 genome editing.

To generate the HCT116 DIMT1L^Y131G/Y131G^, WBSCR22^D82K/D82K^, and SNORD13 KO cell lines, the selected point mutations or deletion of SNORD13 were introduced by CRISPR-Cas9 genome editing on both alleles, as follows: an *in vitro* reconstituted Cas9 RNP complex (final concentration 4 µM) consisting of specific crRNA guides (see Table S2), a universal tracrRNA (IDT, #1072532), and the *Streptococcus pyogenes* Cas9 (IDT, #1081058), was electroporated in cells freshly resuspended in nucleofector solution V (Lonza, VCA-1003) together with a single-strand donor DNA (ssDNA, final concentration 4 µM) in case of the point mutations. Cells were electroporated with an enhancer (IDT, #1075915, final concentration 4 µM) in a nucleofector device (Lonza, Nucleofector 2; programme D-032). Cells were incubated for 24 h to allow them to recover and then detached and cloned by serial dilution. Individual clones were selected and diagnosed by PCR amplification of the modified area (for SNORD13 KO clones, clone #2 was used), followed by differential restriction digest for DIMT1L^Y131G/Y131G^ clones (gain of a *Bst*NI restriction site, clone #10 was used) and WBSCR22^D82K/D82K^ clones (loss of an *Eco*RV restriction site, clone #1 was used) as well as by DNA sequencing of the modified area.

### Cell culture

HCT116 cells were cultured in McCoy’s 5A medium (Lonza, BE12-168F) supplemented with 10% foetal bovine serum (Sigma, F7524), 100 U/ml penicillin, and 100 µg/ml streptomycin (Lonza, DE17-602E) in a New Brunswick Galaxy 170 R incubator at 37°C and under 5% CO_2_.

### Isolation of total RNA

Total RNA from HCT116 cells was extracted in TriReagent solution (Thermo Fisher) according to the manufacturer’s instructions.

### Isolation of genomic DNA

Genomic DNA was isolated from five Mio HeLa cells using the NucleoSpin tissue kit (Macherey-Nagel) according to the manufacturer’s protocol.

### *Generation of templates for* in vitro *transcription*

The complete 18S rRNA sequence was amplified from genomic DNA by touchdown PCR with Q5 DNA polymerase (New England Biolabs) using a forward primer that introduces the T7 promoter sequence for *in vitro* transcription (IVT). The following protocol was used for touchdown PCR: 30 sec initial denaturation at 98°C, 20 cycles of touchdown (10 sec, 98°C; 20 sec, 72°C to 62°C (∆Tm −0.5°C); 5 min, 72°C), followed by 15 cycles standard PCR at 62°C annealing temperature and final elongation (5 min, 72°C). The primer sequences are listed in Table S1.

### In vitro *transcription*

The 18S IVT was generated using the T7 Megascript kit (Thermo Fisher Scientific) according to the manufacturer’s protocol. RNA integrity was analysed on a 1% agarose gel. The IVT product was purified using RNA Clean and Concentrator kit (Zymo Research).

### Polyadenylation of 18S IVT

One-microgram 18S IVT was polyadenylated with an E-PAP based Poly(A) Tailing Kit (Thermo Fisher Scientific) according to the manufacturer’s instructions and purified using RNA Clean and Concentrator kit (Zymo Research).

### Generation of ONT direct RNA-seq libraries for sequencing on FLO-MIN106D (R9.4.1) flow cells

Direct RNA-seq libraries were generated using the SQK-RNA002 kit (Oxford Nanopore Technologies) following the sequence-specific protocol. Universal oligo A and sequence-specific oligo B (Table S1) were annealed at a concentration of 1.4 µM each in 10 mM Tris, pH 7.5, 50 mM NaCl (2 min, 95°C; 0.1°C/sec to 22°C). Briefly, 500 ng total RNA in a volume of 9 µl was ligated to 1 µl custom adapter using 1.5 µl T4 DNA ligase (New England Biolabs) in NEB next Quick ligation buffer (3 µl, New England Biolabs) in the presence of 0.5 µl RNA CS (Oxford Nanopore Technologies) for 10 min at room temperature. Reverse transcription to stabilize the RNA strand was performed using Superscript IV reverse transcriptase (Thermo Fisher Scientific for 50 min at 50°C, followed by enzyme inactivation (10 min, 70°C)). Reactions were cleaned up using Agencourt RNAClean XP beads (Beckman Coulter). The RMX RNA adapter was ligated as described above, followed by Agencourt RNAClean XP purification and elution in 21 µl elution buffer. The concentration of the library was determined using Qubit DNA HS assay (Thermo Fisher Scientific). Libraries were sequenced on a GridION X5 device equipped with MinION R9.4.1 flow cells for 48 h and basecalled with Guppy 5.0.11 in fast-basecalling mode.

### Generation of ONT direct RNA-seq libraries for sequencing on Flongle flow cells

Libraries for sequencing on Flongle flow cells were prepared as above with some modifications. Libraries were prepared with 200 ng total RNA as input. After the first cleanup, RNA was eluted in 10 µl H_2_O and the following steps carried out in a smaller volume: 10 µl RNA were ligated to 2 µl RMX with 1 µl T4 DNA Ligase in a total volume of 20 µl and purified with an equal volume of Agencourt RNAClean XP beads. Libraries were eluted in 9 µl ELB. Flongle flow cells were loaded by a community protocol to allow loading similar to the FLO-MIN106D flow cells (https://community.nanoporetech.com/posts/a-very-gentle-relatively). Flongle flow cells were primed with 117 µl FLB +3 µl FLT. Eight-microlitre library was diluted with 7 µl H_2_O and loaded with 15 µl RRB. Flongle libraries were sequenced on a GridION X5 device equipped with Flongle adapters and Flongle flow cells for 24 h and basecalled with Guppy 5.0.11 (Figure 5: 5.1.13) in fast-basecalling mode.

### Preprocessing of direct RNA-seq data

Reads were mapped using minimap2 version 2.17. BAM files were filtered to exclude secondary and poor alignments. Plus, the MD tag was added to allow variant calling using JACUSA2 software without the need for the reference transcriptome.

### Detection of modifications with JACUSA2

The JACUSA2 software [31] calculates individual scores for Mismatch, Deletion, and Insertion events. We employ the JACUSA2 call-2 run mode throughout this manuscript, which compares replicate samples from two conditions. If not otherwise stated, we use 1,000 reads for our analyses throughout the manuscript. We tested the following feature sets derived from JACUSA2 to take into account the clustering of rRNA modifications, as well as the inherent characteristic of the current nanopore pore protein to cover 5-mers: a) Mismatch score of the analysed site (M), b) Mismatch, Insertion, and Deletion scores of the analysed site (MDI), c) Mismatch score of the 5-mer context (modified site in position 3), Insertion, and Deletion score of the analysed site (M_Con_DI) and d) Mismatch, Insertion, and Deletion score of the 5-mer context ((MDI)_Con_). We either use these JACUSA2 scores and feature sets directly or employ them as input to the Local Outlier Factor (LOF) [42] method. To generate JACUSA2 score plots, our software performed WT vs. IVT comparisons on either one or three replicate samples. In case of the genetic model systems, where we can make an assumption on the number of differential sites, we applied the LOF method which predicts outliers in an unsupervised manner by measuring the density deviation of each point with respect to its neighbours. We predict positions with the highest LOF score as modified. We only compute LOF scores for positions where the JACUSA2 score is above the median of the score distribution. We compute the LOF score for all positions using the set of pairwise comparisons as features. The proportion of outliers to be captured for the analysed cases was set to 0.1–0.2% (contamination value 0.001–0.002), depending on the expected number of differential sites. We used the ‘LocalOutlierFactor’ function from the scikit-learn python package with the default neighbourhood size 20 to compute LOF scores and the matplotlib package for visualization. We labelled the identified outliers as modified site, neighbours (−2 to +2) or non-modified positions *post hoc* based on the analysis by [8].

### rRNA benchmark

The rRNA benchmark has been implemented as a snakemake (v7.25.3) workflow to measure the performance of different software solutions in identifying RNA modifications. All employed tools perform pairwise comparisons where WT and KO/IVT conditions are contrasted. Performance has been measured against a set of known 18S rRNA modifications. The influence of basecalling, and the number of replicates has been investigated by running tools on data sets processed with different combinations of the aforementioned factors. Calculations have been carried out on the same node in a slurm cluster (see Supplementary Text for details). The area under the precision recall curve has been used to compare the performance of the tools (Figure S2, Figure S3 and Supplementary Text). The running times of the tools were determined in triplicates (Table S4, Supplementary Text).

### Downsampling analysis

To evaluate the effect of read coverage on the analysis, BAM files were downsampled to different amounts of reads (0.3k, 0.5k, 1k, 5k, 10k). We employ various seed values for the downsampling procedure. The generated down-samplings were subjected to the JACUSA2 call-2 analysis. To compare results across the different levels of read coverage, we calculated the distance between modification sites and the median in terms of two basic scores: the JACUSA2 Mismatch scores and the score assigned to each site by the LOF method. To avoid bias caused by the different scales of LOF scores across analyses, the normalized distance was considered so that the difference between the score of the modification site and the median is divided by the maximum LOF value.

### Mixing analysis

To evaluate the ability to detect rRNA modifications with low stoichiometry, *in silico* samples with different averages of modification rates (0%, 0.5%, 5%, 10%, 25%, 50%, 75%, 100%) were designed by combining WT and KO/MUT samples of 1,000 reads. Then, differential analysis of the generated mixtures and the MUT/IVT samples was performed using JACUSA2 call-2. For the experimental mixing analysis, WT and KO/MUT RNA were mixed with the indicated ratios and libraries were prepared for sequencing on Flongle flow cells as described above. The JACUSA2 Mismatch score was compared across different mixture ratios.

Preprocessing, down-sampling, and mixing were performed using Samtools version 1.9. A Snakemake pipeline for the analysis workflow was developed and is available on Zenodo (https://doi.org/10.5281/zenodo.8268171).

### Northern blot

The loss of SNORD13 in HCT116 SNORD13 KO was analysed by Northern blotting [43] using a probe described in Table S2. Ethidium bromide staining was used to control loading.

### Primer extension assay

Primer extension assays were used to validate the loss of specific 18S rRNA modifications in CRISPR-Cas9-engineered HCT116 cell lines as described previously [38,44,45] using 2 µg total RNA and primers listed in Table S3.

### Misincorporation assay

Acetylation of 18S rRNA C_1842_ was analysed by NaCNBH_3_ reduction, followed by TGIRT-III reverse transcription and Sanger sequencing of the PCR product based on the method described [45–47]. Briefly, 200 ng total RNA was reduced with 100 mM NaCNBH_3_ in 100 mM hydrochloric acids for 20 min at room temperature in a total volume of 100 µl. Reactions were quenched by the addition of 30 µl 1 M Tris pH 8.0 and purified with RNA Clean & Concentrator-5 kits (Zymo Research). 200 pg reduced RNA or non-treated control (not shown) was reverse transcribed with a primer targeting the H45 of the 18S rRNA (18S H45 rev) and TGIRT-III reverse transcriptase. RNA was mixed in a total volume of 17 µl with 4 µl 1 µM RT primer and 4 µl 5 × TGIRT buffer (2.25 M NaCl, 25 mM MgCl_2_, 100 mM Tris pH 7.5) and incubated 3 min at 75°C, followed by 3 min on ice. Then, 1 µl 100 mM DTT and 0.5 µl TGIRT-III (Ingex) were added. Reactions were incubated for 20 min at room temperature. After addition of 1 µl dNTP mix with reduced dGTP (10 mM dATP, dTTP, and dCTP and 5 mM dGTP), reactions were incubated 60 min at 57°C. PCR reactions were composed of 2 µl cDNA, 2.5 µl 10 µM 18S H45 forward and reverse primer, respectively, 10 µl 5 × HF buffer, 1 µl 10 mM dNTPs, 1 µl Phusion Hot Start Flex DNA Polymerase (New England Biolabs) and 31 µl water. Reactions were cycled with the following programme: 30 sec initial denaturation at 98°C; 35 cycles (10 sec, 98°C; 15 sec, 67.4°C; 15 sec, 72°C) and final elongation (5 min, 72°C). PCR products were analysed on 2% agarose gels stained with GelRed (Biotium) and cleaned up with NucleoSpin Gel and PCR Clean-up Kit (Macherey-Nagel). PCR products were analysed by Sanger sequencing (LGC Genomics) with the 18S H45 forward primer. Primer sequences used in the Misincorporation assay are listed in Table S3. Chromatograms were analysed with Chromas 2.6.6 (Technelysium Pty Ltd).

## Results

### Targeted direct ribosomal RNA-seq in total human RNA samples

**Figure 1.**
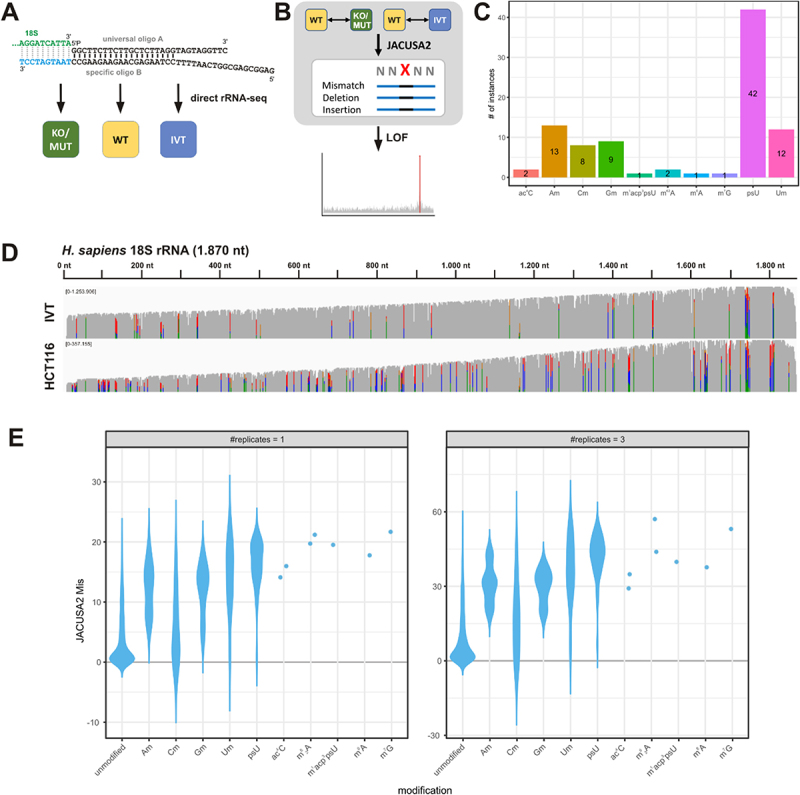
Setup of targeted direct rRNA sequencing. (A) a custom adapter is employed for sequencing of the human 18S rRNA (direct rRNA-seq) in samples representing different modification status: wild type (WT), knock out/mutant (KO/MUT) or *in vitro* transcribed rRNA (IVT). The custom adapters consist of an universal oligo A annealed to a sequence specific oligo B. The sequence-specific part of oligo B (light blue) has a length of 10 nts and anneals with the 3′ end of the 18S rRNA (green). (B) Overview of the analysis workflow. BAM files from the direct rRNA-sequencing (A) are compared pairwise employing JACUSA2 call-2, which calculates scores for different error profiles (Mismatch, Deletion, Insertion). These scores can be combined to feature sets, taking only the target site, or as well the 5-mer context into account. Significant outliers are identified by Local outlier Factorization (LOF). (C) Abundance of modification types on the 18S rRNA. (D) Coverage of nanopore direct rRNA-seq of 18S IVT and 18S rRNA from HCT116 WT cells sequenced on MinION R9.4.1 flow cells. Allele frequency threshold = 0.2. Mismatches are indicated by IGV default colours. (E) Violin plot summarizing the JACUSA2 call-2 analysis of the 18S rRNA from HCT116 WT cells and 18S IVT. Shown is the JACUSA Mis score for all modification types on fast basecalled data as indicated. Left panel: analysis of a single replicate, right panel: analysis of three replicates.

The direct analysis of rRNA sequence variants and of rRNA modifications has only become possible recently, thanks to the advent of the direct RNA-sequencing (direct RNA-seq) platform developed by Oxford Nanopore Technologies (ONT). Here, we established a protocol with minimal pre-processing to enable the analysis of low-input samples (Figure 1A,B). To prevent the laborious purification of individual rRNAs, which often suffers from material loss and introduction of biases, or additional experimental steps, such as *in vitro* polyadenylation, we established a custom adapter (analogous to [48]) for the selective sequencing of human 18S rRNA that captures the 3′ end of the mature rRNA for direct rRNA-sequencing (Figure 1A). Input for our analysis was BAM files (basecalling with Guppy, alignment with minimap2) sampled to equal read numbers (1,000 or 5,000 reads). These were then subjected to a Snakemake workflow for pairwise JACUSA2 call-2 analysis. JACUSA2 calculates scores for different basecalling errors: Mismatch, Deletion, and Insertion, which can be combined to feature sets. Identification of significantly different sites may be formulated as an outlier detection problem with local outlier factorization (LOF) (Figure 1B). The human 18S rRNA harbours 91 modification sites, with pseudouridines (psU) and 2′-O-ribose methylation (Nm) being the largest classes (Figure 1C).

First, we compared the performance of two sequence-specific adapters with different lengths to the standard oligo(dT) adapter (RTA) employing an *in vitro* transcribed (IVT) 18S rRNA. A ten nucleotides long adapter was sufficient to efficiently capture the 18S rRNA (Figure S1A,B). Furthermore, this analysis revealed sequencing of incomplete (missing 3′ end) and reverse strand RNA molecules with the standard RTA. This problem was not observed with the sequence-specific adapter (Figure S1D-E). This adapter was then used for direct rRNA-seq of rRNA from human HCT116 cells and compared to the sequencing of a full-length 18S IVT (Figure 1D). The cellular rRNA displayed a higher mismatch rate than the IVT (coloured lines, Figure 1D), which is indicative of the presence of rRNA modifications detectable *via* basecalling errors with the JACUSA2 algorithm [31].

To elucidate whether JACUSA2 is indeed suitable to identify well-defined rRNA modifications [8], we analysed the 18S rRNA from HCT116 wild-type cells (WT) and the corresponding IVT using JACUSA2 call-2 (WT vs. IVT) and calculated Mismatch (Mis), Deletion and Insertion scores. Furthermore, we considered the 5-mer context (as the R9.4.1 nanopore protein covers five nucleotides at a time) to test whether it contributes to the modification signal. This results in four feature sets that were derived from JACUSA2 as described in the Methods section.

The performance of these JACUSA2 feature sets was benchmarked against other tools designed for the identification of RNA modifications by direct RNA-seq, namely xPore [34], Nanocompore [36], EpiNano [28,32,35] and Eligos2 [30]. We noticed that JACUSA2, also on replicate samples, and EpiNano have the shortest run times (Table S4). All tools were run on fast and high-accuracy (HAC) basecalled data. The AUC for precision and recall was calculated for all modifications and additionally stratified according to modification type (pseudouridine, 2′-O-ribose methylation and other modifications) for one and if possible three replicates (Figure S2). For all modifications, JACUSA2 Mis, EpiNano linear and EpiNano delta had the best performance, with EpiNano being slightly better on HAC basecalled data (Figure S2, panel 1). Interestingly, the performance of many tools was strongly dependent on the modification type. Here, EpiNano was best in detecting 2′-O-ribose methylation sites (Figure S2, panel 3), whereas JACUSA2 Mis and JACUSA2 MDI were superior in detection of diverse ‘other’ modifications (Figure S2, panel 4). We further stratified the 2′-O ribose methylation sites according to the nucleobase (Figure S3). This reveals that in case of Cm the 5-mer context and the use of replicates are important for modification calling (Figure S3, panel 2). However, as the Mis score is the main determinant for all other modification types, we decided to use JACUSA2 Mis on fast basecalled data throughout this study.

The JACUSA2 Mis scores for the WT – IVT comparison were further stratified for every modification type (Figure 1E), revealing the capability to detect ac^4^C, m${\;}_{2}^{6}A$, m^1^acp^3^psU, m^6^A and m^7^G in nanopore direct RNA-seq data. JACUSA2 Mis scores on HAC basecalled data (Figure S4) and for the other JACUSA2 feature sets (Figure S5) are provided in the Supplementary Material.

In conclusion, Nanopore direct rRNA-seq coupled to JACUSA2 analysis is suitable for the detection of diverse RNA modifications in human rRNAs and may be especially useful for detecting base modifications as methylation and acetylation.

### Nanopore direct rRNA-seq enables the detection of site-specific RNA modifications

Modification sites on rRNAs, including human rRNAs, have been characterized extensively by a range of techniques, including classical RNA biochemistry (such as primer extension), HPLC, mass spectrometry, short-read deep sequencing-based methods (see Introduction), and more recently, by CryoEM. For most of these modifications, the responsible enzymes and, when relevant, the antisense snoRNA guides have been identified and characterized. We made use of this knowledge to analyse specific rRNA base methylations of the 18S rRNA in human cells lacking individual modifications by targeted nanopore direct RNA-seq as a proof-of-concept.

**Figure 2.**
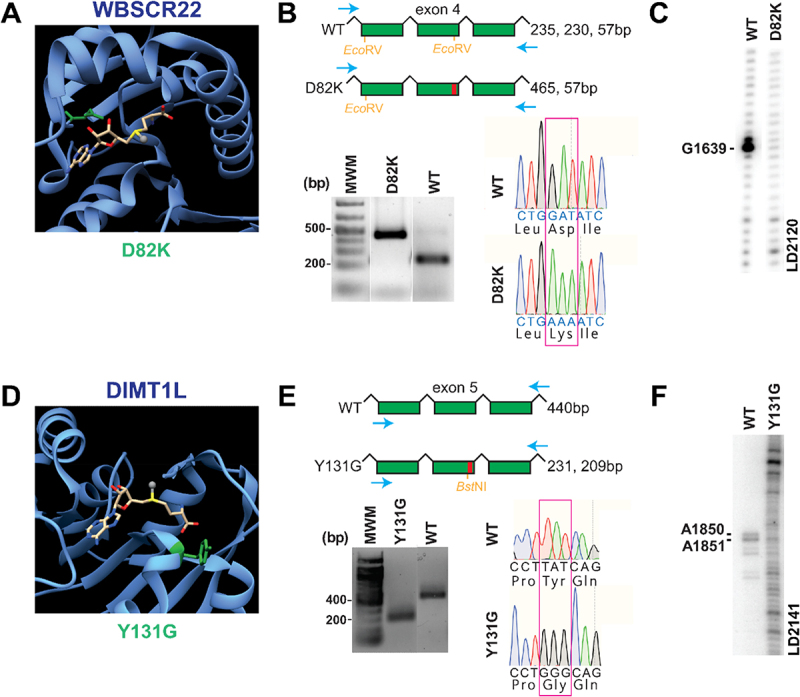
Generation and validation of human cell lines expressing a catalytically inactive form of the 18S rRNA methyltransferases WBSCR22 or DIMT1L. A mutation encoding a single amino-acid substitution in the catalytic pocket of the methyltransferase (Y131G for DIMT1L, D82K for WBSCR22), was introduced on both alleles of HCT116 cells by CRISPR-Cas9 genome editing. (A) 3-D model based on the yeast homolog of WBSCR22, BUD23 (PDB 4QTU). The mutated residue is highlighted in green in the catalytic pocket of the enzyme. The methyl donor cofactor, *S*-adenosyl methionine (SAM), is depicted in stick representation with the methyl group to be transferred as a grey sphere. (B) Diagnostic evaluation by differential restriction of PCR fragments, and by DNA sequencing. The PCR products were amplified from genomic DNA extracted from the mutant cell line and the isogenic control (WT). The primers used (blue arrows) and the size of the expected fragments upon *Eco*RV digestion are shown. Red box, catalytic domain. Restriction digests were analysed by agarose electrophoresis followed by ethidium bromide staining. DNA sequencing profiles at the mutation site are shown. (C) Loss of RNA modification in the mutant cell line was confirmed by primer extension assay performed with oligo LD2120 on total RNA cleaved at m^7^G following treatment with NaBH_4_ and aniline as in [52]. (D) 3-D model based on human DIMT1L (PDB 1ZQ9), coloured elements as in A. (E) Diagnostic evaluation of differential restriction of PCR fragments after *Bst*NI digestion as in B. (F) For DIMT1L, the primer extension was performed on total RNA with oligonucleotide LD2141 as in [9]. Loss of modification was further confirmed by HPLC analysis (data not shown).

For this, we generated human colon carcinoma-derived cell lines (HCT116) genetically engineered by CRISPR-Cas9 genome editing to harbour either a knock-out (KO) or a catalytic-dead variant (MUT) of selected methyltransferases (see Material and Methods for details). The cell lines and affected modifications are listed in Table 1. The METTL5 KO cell line was described previously, and it lacks the m^6^A modification at position 1832 of the human 18S rRNA [37]. In addition, we generated cell lines that express catalytic dead variants of DIMT1L and WBSCR22 (Figure 2). DIMT1L catalyses the double m${\;}_{2}^{6}A$ modification at positions 1850 and 1851 of the 18S rRNA [9,38,39], whereas WBSCR22 catalyses the m^7^G modification at position 1639 of the 18S rRNA [9,40,41]. The conserved D82 amino acid in the catalytic pocket of WBSCR22 (Figure 2A) was mutated to a lysine, resulting in loss of a diagnostic *Eco*RV site as monitored by differential restriction digest (Figure 2B). As expected, in cells expressing the WBSCR22 D82K variant, the methylation of G_1639_ was no longer detected in a primer extension assay (Figure 2C) following NaBH_4_-aniline treatment [44]. In case of DIMT1L, the Y131 residue, located in the catalytic pocket of the enzyme, was substituted by a glycine (Figure 2D). The introduction of the mutation resulted in the gain of a *Bst*NI restriction site (Figure 2E). A primer extension assay revealed that the DIMT1L Y131G variant led to loss of the A_1850/1851_ double dimethylation (Figure 2F), a result which was further confirmed by HPLC analysis (data not shown). Note that the double m${\;}_{2}^{6}A$ modification is rather ‘bulky’ and is naturally causing a reverse transcription drop-off. For both WBSCR22 and DIMT1L, the bi-allelic knock-in was confirmed by DNA sequencing of the region of interest (Figure 2B,E).

**Table 1.** Genetically engineered HCT116 cell lines analysed by direct rRNA-seq.

Cell line	Missing modification	Reference modification enzyme
HCT116 WT	-	
HCT116 WBSCR22^D82K/D82K^	18S m^7^G_1639_	[9,40,41]
HCT116 METTL5^−/−^	18S m^6^A_1832_	[37,49]
HCT116 DIMT1L^Y131G/Y131G^	18S m${\;}_{2}^{6}A$_1850_ m${\;}_{2}^{6}A$_1851_	[9,38,39]
HCT116 SNORD13^−/−^	18S ac^4^C_1842_	[43,50,51]

**Figure 3.**
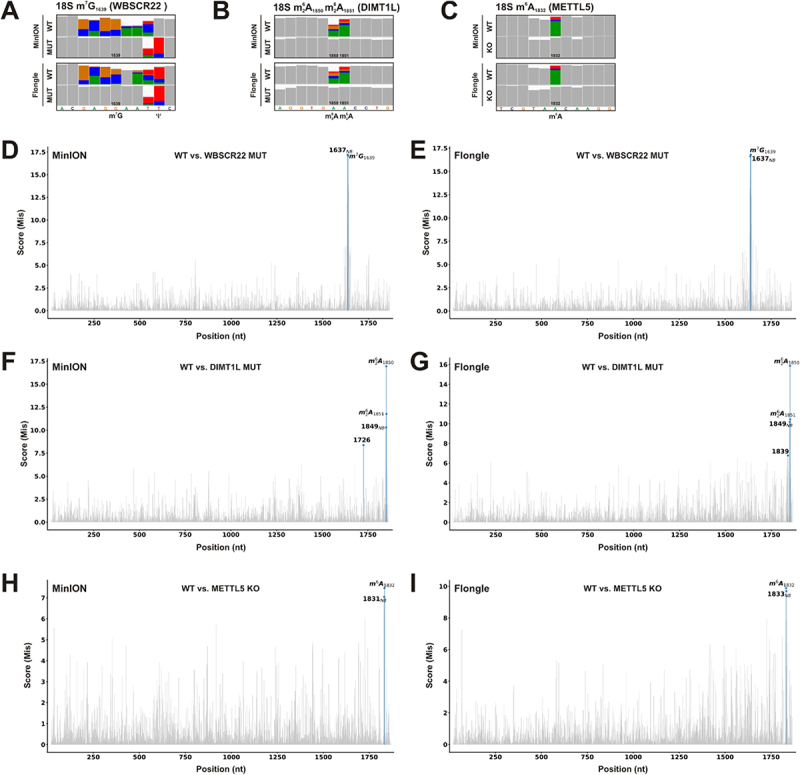
Detection of site-specific modifications in genetically engineered HCT116 cells by nanopore direct rRNA-seq and JACUSA2 call-2. HCT116 cells as listed in Table 1 were subjected to nanopore direct rRNA-seq, either on a MinION or Flongle flow cell as indicated. (A-C) IGV snapshots of the regions of interest from MinION or Flongle sequencing as indicated. The target site as well as other described modifications are annotated. Allele frequency threshold = 0.2. (D,F,H) Barplots of the pairwise comparisons of MinION derived data by JACUSA2 call-2 analysis considering the Mismatch score. Significant outliers detected by Local outlier Factorization (contamination value = 0.001 for WBSCR22 and METTL5, 0.002 for DIMT1L) are labelled in blue. Outliers in the 5-mer context of the target site are marked by ‘NB’. (E,G,I) Barplots of the pairwise comparisons of Flongle derived data, as in D,F,H. D,E) analysis of 18S m^7^G_1639_ employing HCT116 WBSCR22^D82K/D82K^. (F,G) analysis of 18S m${\;}_{2}^{6}A$_1850_ m${\;}_{2}^{6}A$_1851_ employing HCT116 DIMT1L^Y131G/Y131G^. (H,I) analysis of 18S m^6^A_1832_ employing HCT116 METTL5^−/−^.

These cell lines were employed for pairwise comparisons with the isogenic WT control (Figure 3). We detected characteristic base calling errors in the WT control in all analysed cases as higher Mismatch frequencies in IGV snapshots (Figure 3A–C) in comparison to WT samples. However, different modification types affected base calling differently. Strikingly, m^7^G_1639_ affected basecalling not only at the actual modified position but also strongly at the neighbouring residues (−3 to +3), providing a powerful signature (Figure 3A). On the other hand, the m^6^A and m${\;}_{2}^{6}A$ modifications resulted mainly in basecalling errors at the target site (Figure 3B,C).

The pairwise comparison of WT and KO/MUT cell lines with JACUSA2 call-2 and 1,000 reads per condition revealed high JACUSA2 Mis scores for the target sites, but not for non-related positions (Figure 3D-I). Here, we considered modifications as outliers (i.e. unusual JACUSA2 scores within a set of positions) employing the previously introduced Local Outlier Factorization (LOF) [42] to rank positions by their degree of outlierness (see Methods). The LOF approach has two hyperparameters: neighbourhood size and contamination. The contamination value determines the proportion of points with the highest LOF scores to be called as outliers. As we expect to find only one or two outliers, we applied here strict contamination values of 0.001 (WBSCR22, METTL5) and 0.002 (DIMT1L), respectively, which corresponds to 0.1% and 0.2% of sites. Strikingly, for all analysed modifications, the target site as well as one to two adjacent sites were identified as significant outliers (blue) (Figure 3D-I).

Here, we used the JACUSA2 Mis score to call modification sites. However, we would like to note that depending on the data and modification type, also the Deletion and Insertion score as well as the 5-mer context may improve modification calling (Table S5).

Current input requirements may preclude the use of nanopore direct RNA-seq for samples with limited availability. Furthermore, multiplexing of samples with barcoding is currently not officially supported for direct RNA-seq by ONT, increasing the cost for direct RNA-seq experiments. To overcome these problems, we aimed to transfer the targeted direct rRNA-seq approach described above from the standard MinION flow cells (512 channels with 4 pores each) to the recently introduced smaller Flongle flow cells (126 pores), which require less RNA input. The overall results of the JACUSA2 analysis were highly comparable for MinION and Flongle flow cell-derived data (Figure 3 compares panels D, F, and H with E, G, and I). Also, the IGV snapshots for the analysed modification sites were remarkably comparable between MinION and Flongle data (Figure 3A–C, compare upper and lower panels).

In summary, all analysed base methylation sites on the 18S rRNA were detected with JACUSA2 in the MinION flow cell data as well as in the Flongle flow cell data. In addition, the generated mismatch profile was highly similar between MinION and Flongle sequencing. Overall, the Flongle-based approach enables the analysis of low input samples such as patient-derived material making it amenable to clinical biology.

### The optimal coverage is determined by the modification type

To exclude the impact of coverage on our analysis, we used 1,000 reads throughout this work to analyse differential RNA modifications (Figure 3). To elucidate whether a higher number of reads would be beneficial for the analysis, or if an even lower number of reads may be useful, we sampled different read numbers from our MinION datasets. As expected, the difference between the JACUSA2 score of the target site and the median JACUSA2 score increases, when more reads are considered for analysis (Figure S6A-C, left panels). To evaluate the robustness of outlier identification, we calculated the normalized distance of the target site LOF scores to the median LOF score (Figure S6A-C, right panels). Surprisingly, we noticed a small decrease in the normalized LOF score distance for WBSCR22 (Figure S6A, right panel), which was, on the other hand, accompanied by a decrease in the standard deviation at higher read numbers. Robustness in the detection of the METTL5-catalysed m^6^A modification was increased by higher read numbers, as indicated by the increased normalized LOF score distance and decreased standard deviation at 5,000 and 10,000 reads, respectively (Figure S6C, right panel). For the DIMT1L target sites, the identification is mostly independent of the number of analysed reads (Figure S6B). In summary, 1,000 reads are sufficient to detect the analysed modifications. In most cases, more reads are not beneficial and less reads are sufficient.

### Nanopore direct rRNA-seq is suitable to estimate modification levels

**Figure 4.**
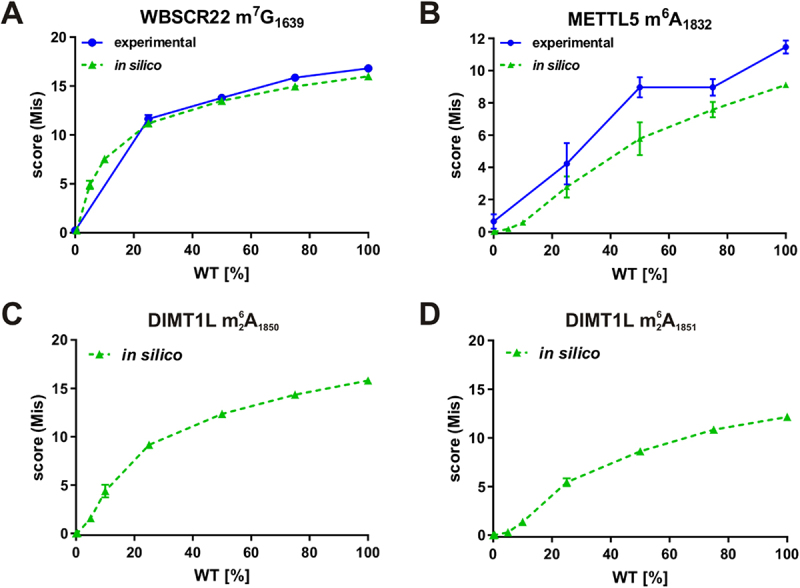
Analysis of the influence of modification levels on the JACUS2A score determined by *in silico* or experimental mixing analysis. For the *in silico* mixing analysis 1,000 reads were downsampled from the MinION sequencing data shown in Figure 3. The ‘Mix’ sample was composed of modified (WT) and unmodified (KO/MUT) reads as indicated that were derived from the downsampled data with 5 different seeds. For the experimental mixing analysis, RNA from WT and WBSCR22 MUT (A) or METTL5 KO (B) cell lines was mixed in the indicated amounts and subjected to direct rRNA-seq on Flongle flow cells. As for the *in silico* mixing, they were analysed with 1,000 sampled reads. JACUSA2 call-2 analysis considering the Mismatch score. (A) Analysis of 18S m^7^G_1639_ in HCT116 WT and WBSCR22 MUT cells. (B) Analysis of 18S m^6^A_1832_ in HCT116 WT and METTL5 KO cells. (C,D) analysis of 18S m${\;}_{2}^{6}A$_1850_ or m${\;}_{2}^{6}A$_1851_, respectively, employing HCT116 WT and DIMT1L MUT cells.

As described above, in a clear cut situation, when a cell line harbouring a knock-out (KO) or catalytically dead variant (MUT) of an enzyme is compared to a wild-type cell line (Figure 3, Figure S6), 1,000 reads were well suited for the analysis of differential RNA modifications. However, in physiological or pathological contexts, more subtle changes in modification levels are often expected. We were therefore interested to learn to what extent rRNA modifications could also be analysed at substoichiometric levels with our approach. We approached this question both experimentally and *in silico* by mixing either RNA or sequencing reads from WT and KO/MUT at various ratios. We first sampled *in silico* a total of 1,000 reads from all samples. For the reference ‘Mix’ sample, different ratios of WT and KO/MUT reads were bioinformatically mixed as indicated (Figure 4A–D). Five replicate samples from all mixing ratios were analysed by pairwise JACUSA2 comparison as outlined above. Interestingly, m^7^G_1639_ and m${\;}_{2}^{6}A$_1850/1851_ were consistently detected with only 5–10% modified reads, whereas m^6^A_1832_ had a detection threshold of around 25%. Importantly, an increase in the JACUSA2 score with increasing modification frequency was detected for all analysed modification sites (Figure 4A–D), indicating that nanopore direct rRNA-seq can also be used for estimation of modification levels. For the m^7^G and m${\;}_{2}^{6}A$_1850_ modifications, the JACUSA2 score seems to approach saturation (Figure 4A,C).

To validate these findings, experimental mixing analyses were performed with WBSCR22 MUT and METTL5 KO RNAs (Figure 4A,B). Importantly, the results of the experimental mixing analyses were comparable to the *in silico* mixing analyses. For both analysed modification types, the JACUSA2 score increases with increasing modification levels and approached saturation at higher modification levels (Figure 4A,B). In conclusion, we show that nanopore direct rRNA-seq in combination with JACUSA2 analysis can be used to estimate modification levels providing an appropriate calibration curve is established that may also be generated *in silico*.

### Beyond methylation – detection of acetylcytidine

**Figure 5.**
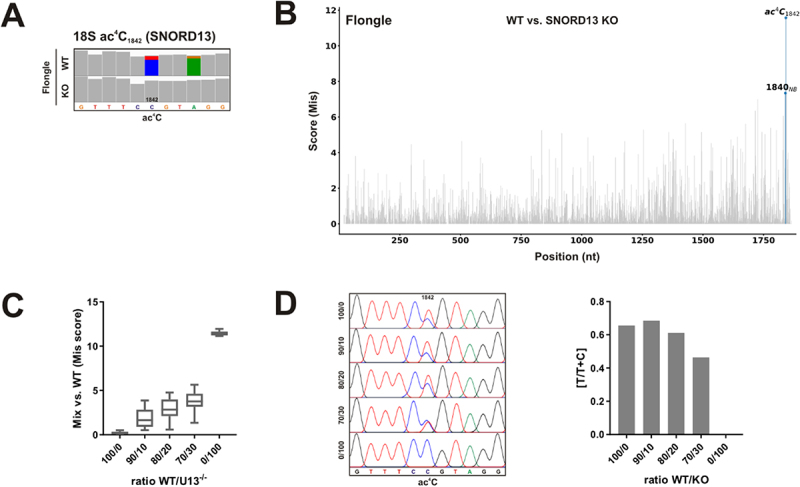
Analysis of 18S ac^4^C_1842_ by nanopore direct rRNA-seq and an orthogonal method employing HCT116 SNORD13 KO cells. (A) IGV snapshots of the region of interest from HCT116 WT and SNORD13 KO cells as indicated. The target site is annotated. Allele frequency threshold = 0.18. (B) Barplots of the pairwise JACUSA2 call-2 analysis. Significant outliers detected by LOF (contamination value = 0.001) are labelled in blue. JACUSA2 Mismatch score was considered. (C) JACUSA2 Mis scores of pairwise comparisons of different experimental RNA mixes against the HCT116 WT RNA. Reads were downsampled to 1,000 reads with different seeds (*n* = 15). (D) 18S helix 45 ac^4^C misincorporation assay based on NaCNBH_3_ reduction and TGIRT-III reverse transcription for RNA mixtures as indicated. Left panel: Sanger sequencing traces. Right panel: quantification of the C-to-T misincorporation.

In human cells, NAT10, which is essential for pre-18S rRNA processing, works together with SNORD13 (U13) to install the acetylation of 18S at position C_1842_ [43,50,51], which is one of the two ac^4^C modifications found on the 18S rRNA. We generated an HCT116 SNORD13 KO cell line by CRISPR-Cas9 genome editing (Figure S7A,B). The loss of C_1842_ acetylation in this cell line was validated by a primer extension assay (Figure S7C) and direct rRNA-seq – JACUSA2 analysis that identified ac^4^C_1842_ as a *bona fide* outlier (Figure 5A,B).

As the identification of small changes in rRNA modification levels is presumably of biological and clinical interest, we compared our approach to an orthogonal method, namely a C-to-T misincorporation assay based on borohydride reduction [45–47]. To simulate a scenario of small changes in acetylation, we analysed WT/SNORD13 KO mixtures with only 10%, 20%, and 30% of KO RNA (Figure 5C,D). Nanopore direct rRNA-seq revealed a gradual increase of JACUSA2 scores in pairwise comparison to the WT RNA (Figure 5C). Interestingly, JACUSA2 is particularly sensitive to relatively small changes in C_1842_ acetylation. On the other hand, the C-to-T misincorporation assay could not discriminate between 100% WT and 90% WT/10% SNORD13 KO, revealing an inherent limitation of this approach (Figure 5D).

We therefore concluded that nanopore direct rRNA-seq can detect even small changes in modification levels, whereas the misincorporation assay is only suitable to detect larger differences.

In summary, we established the nanopore-based targeted direct RNA-sequencing of human ribosomal RNAs and analysed widespread rRNA modifications employing JACUSA2. We show that direct rRNA-seq on the nanopore can be scaled down to the Flongle device, enabling the analysis of clinical or biological samples with limited availability.

## Discussion

Modifications of human rRNAs have been characterized for many years, and until recently were considered as relatively constitutive. Emerging evidence however supports the hypothesis of ribosomal heterogeneity [53], which includes the production of differentially modified ribosomes that may contribute to the aetiology and progression of several diseases including cancer, developmental and cardiovascular disorders.

Thus, there is a need to develop techniques for the in-depth characterization of the entire rRNA modification repertoire in a quantitative fashion. Ideally, such techniques should be amenable to low input material and high throughput analysis.

Consequently, the sequence specific and quantitative analyses of various RNA modification types with moderate amounts of input material are progressing fast with the advent of the direct RNA-sequencing method. However, current analyses have focused mostly on highly specific types of RNA modifications as m^6^A and pseudouridine [28,30,31,34–36], or did not address dynamic changes in human samples with clinical relevance [32,33].

Here, we established the targeted direct RNA-seq of human ribosomal RNAs with a selection step involving the use of custom adapters (direct rRNA-seq). Our targeted approach ensures that only properly processed rRNAs with defined 3′ ends are sequenced [54–56], and no unfaithfully processed RNAs (Figure S1). We benchmarked our JACUSA2-based approach against other tools designed to detect RNA modifications in direct RNA-seq data and demonstrate that JACUSA2 may be considered especially for the detection of pseudouridines and the diverse class of ‘other’ modifications (Figure 1E, Figure S2), whereas the detection of 2′-O-ribose methylation sites by different tools differs substantially between the different nucleobases (Figure S3). In future work, there will be a need to develop more sophisticated models that also consider, for example, the context. Based on the comparison of HCT116 WT and IVT samples, we identified almost all known modification sites on the basis of positive JACUSA2 Mis scores (Figure 1E). This validates the general ability of JACUSA2 to identify differential rRNA modifications of all types (pseudouridine, 2’-O methylation of ribose, m^1^acp^3^psU, ac^4^C, m^7^G, m^6^A, m${\;}_{2}^{6}A$ on the 18S rRNA). However, the main application of JACUSA2 and the comprehensive workflow presented here is the comparative analysis of biological or clinical samples that differ in a limited number of modification sites. In line with previous findings [31,32], we show that Mismatches are the main determinant for the identification of modification sites (Figure 1E). In specific cases, for example Cm modifications, also the Deletion and Insertion scores as well as the 5-mer context may be considered (Figure S5). The rational of taking the 5-mer context into account is based on the fact that the current nanopore protein (R9.4.1) covers five nucleotides at a time. This implies that in case of an RNA modification, also the basecalling at neighbouring sites may be altered.

It is important to emphasize that our prediction of modification sites with JACUSA2 does not rely on any prior knowledge, as we only use modification maps (such as those established experimentally by mass spectrometry by Taoka *et al*. [8]) for *post hoc* evaluation of our results. As for every nanopore error-profile-based approach, the chemical nature of *de novo* identified RNA modification sites needs to be determined by other methods.

Employing a collection of genetically engineered HCT116 cell lines lacking individual modifications (Figure 2), we demonstrated that the recently introduced JACUSA2 software [31] detects all targeted 18S rRNA base methylations in sequencing data produced with the standard MinION flow cells, namely m^7^G_1639_, m^6^A_1832_, and the double dimethylation m${\;}_{2}^{6}A$_1850_ m${\;}_{2}^{6}A$_1851_. These base modifications cause distinct basecalling errors that are reflected by the Mismatch scores calculated by JACUSA2 (Figure 3). Importantly, we could detect rRNA modifications just as efficiently with the small-scale Flongle flow cells (Figure 3), enabling the analysis of precious material available only in limited amounts. This is of particular interest as barcoding of direct RNA-seq libraries is currently not officially supported by Oxford Nanopore Technologies. Sequencing on the Flongle flow cells yields data with comparable quality. All base methylations were identified as significant outliers in this analysis based on 1,000 reads employing the LOF method. The LOF method determines sites that are significantly different from their neighbours as outliers, thus we recommend it for outlier detection when the expected number of differential sites is known or expected to be small. In case the number of differentially modified sites cannot be estimated *a priori*, the contamination value (which defines how many sites are identified as outliers), can be fitted automatically by sklearn.neighbours.LocalOutlierFactor, as described [42]. We do not recommend the LOF method for cases with a high number of differential sites as the WT vs. IVT comparison. Here, we recommend to identify candidate sites based on the JACUSA2 score (see Figure 1E). By downsampling analyses of different read numbers from the MinION data, we show that, remarkably, as little as 300 to 500 reads were sufficient to identify the analysed modification sites (Figure S6). With exception of the METTL5-catalysed m^6^A modification, more reads did not improve results.

We show that the relative modification levels of selected base modifications can be estimated from the JACUSA2 scores based on a calibration curve generated experimentally or *in silico* (Figure 4). In most analysed cases, the JACUSA2 score approaches saturation at higher modification levels (Figure 4A–D); thus, an estimation of modification levels should always be based on an appropriate calibration curve. Only in case of METTL5-catalysed m^6^A_1832_, we oberserved some offset between experimental and *in silico* mixing data (Figure 4B). We speculate that this may be caused by slight variations of the 18S m^6^A_1832_ level in the different biological replicates of HCT116 WT samples used.

Besides the different base methylation sites, the human 18S rRNA harbours in addition two acetylated cytidines. To further expand the repertoire of modifications that may be analysed by nanopore direct rRNA-seq and JACUSA2, we made use of an HCT116 SNORD13 KO cell line. SNORD13 works together with NAT10 to install 18S ac^4^C_1842_ [43,50,51]. Importantly, ac^4^C_1842_ was identified as a *bona fide* outlier in the comparison of SNORD13 KO cells to HCT116 WT cells (Figure 5A,B). Nanopore direct rRNA-seq only requires small amounts of input material and is suitable to identify changes in different modification types at a time. However, we were as well interested in the sensitivity of the method to identify also small changes in modifications that may occur in samples with biological or clinical relevance. To this end, we compared JACUSA2 to an orthogonal validation method based on borohydride reduction of ac^4^C and subsequent reverse transcription misincorporation (Figure 5D). Interestingly, mixture experiments with only small amounts of KO RNA, revealed that direct rRNA-seq – JACUSA2 is particularly sensitive to small changes in RNA modification (Figure 5).

Although we did not cover the complete repertoire of rRNA modifications by dedicated KO cell lines at the time, our data suggest that our workflow captures many if not all rRNA modifications through basecalling error (Mismatch, Insertion, Deletion) analysis.

In this work, we applied JACUSA2 call-2 in pairwise comparisons to identify differences in rRNA modification. A number of other computational pipelines are already available in the literature to analyse rRNA modifications in nanopore direct RNA-seq data and were compared here to JACUSA2 (Figure S2), including Nanocompore [36], EpiNano [35], xPore [34], and Eligos2 [30], which are either based on the detection of basecalling errors, such as in the case of JACUSA2, or which infer modification pattern from changes in the current signal traces. A recently introduced approach, based on signalAlign, estimates modification probabilities on a single read level, but is limited to and only looks at previously described modification sites [33]. Furthermore, some methods are until now only established for specific modification types as m^6^A or pseudouridine [28,32]. The major challenge in the analysis of RNA modifications compared to DNA modifications is the diversity of modification types. Additionally, the expression of different rRNA variants may complicate the analysis.

Importantly, JACUSA2 does not depend on prior knowledge or on training of complex models to identify modification sites. However, in this work, we took advantage of the accurate mapping of rRNA modification sites by mass spectrometry for *post hoc* validation of our findings. In contrast to some other workflows, JACUSA2 supports the handling of replicate samples as well as pairwise comparison [31], enabling either the comparison to an unmodified reference sequence or the identification of differences between biological and clinical samples. JACUSA2 can be used with both Guppy basecalling modes (fast vs. HAC), but of course all analysed samples should be basecalled identically. For the detection of methylation and acetylation, we recommend the fast basecalling mode (Figure S2). We show here that JACUSA2 is not limited to specific modification types but can potentially be used to map and quantify all modification types that cause basecalling errors (Mismatch, Deletion, Insertion). Thus, JACUSA2 could be applied also to the detection of new modification sites, whose chemical nature could be later determined by other methods. It is noteworthy to mention that when we performed comparative analysis we realized that JACUSA2 was also less demanding on computing time than many other algorithms available to date (Table S4).

In conclusion, we established a targeted nanopore direct RNA-seq strategy for human rRNA and detection of modifications by JACUSA2. The down scaling to Flongle flow cells enables the study of samples with limited availability. Future work will focus on the identification of differential modification sites in samples of biological interest and clinical relevance and to understand the biological consequences of altered modification patterns.

## Supplementary Material

Supplemental Material

## Data Availability

Sequencing data generated for this study are available at NCBI’s Sequence Read Archive through the BioProject accession number PRJNA781102. A Snakemake pipeline for the analysis workflow was developed and is available on Zenodo (https://doi.org/10.5281/zenodo.8268171).
