# Supplementary material for: Comprehensive identification of diverse ribosomal RNA modifications by targeted nanopore direct RNA sequencing and JACUSA2
Source: RNA Biol. 2023 Aug 27;20(1):652–65. doi: 10.1080/15476286.2023.2248752 (PMC10464549; doi:10.1080/15476286.2023.2248752)
Supplement: Supplemental Material [file KRNB_A_2248752_SM3150.zip › RNABiology_Supplement_v3_1_.docx]

**Supplementary Information**

**Comprehensive identification of diverse ribosomal RNA modifications by targeted Nanopore direct RNA sequencing and JACUSA2**

Isabel S. Naarmann-de Vries^1,2,+^, Christiane Zorbas^3,+^, Amina Lemsara^1^, Michael Piechotta^1^, Felix G.M. Ernst^3^, Ludivine Wacheul^3^, Denis L.J. Lafontaine^3,§^, Christoph Dieterich^1,2,§,*^

^1^Section of Bioinformatics and Systems Cardiology, University Hospital Heidelberg, Heidelberg, Germany, ^2^German Center for Cardiovascular Research (DZHK), Partner site Heidelberg/Mannheim, Germany, ^3^RNA Molecular Biology, Université libre de Bruxelles (ULB), Fonds de la Recherche Scientifique (F.R.S./FNRS), Biopark Campus, 6041 Gosselies, Belgium

^+^These authors contributed equally to this work.

^§^These authors contributed equally to this work.

^∗^corresponding author: [christoph.dieterich@uni-heidelberg.de](mailto:christoph.dieterich@uni-heidelberg.de)

Author e-mail adresses:

[isabel.naarmann-devries@uni-heidelberg.de](mailto:isabel.naarmann-devries@uni-heidelberg.de)

[christiane.zorbas@ulb.be](mailto:christiane.zorbas@ulb.be)

[lemsaraamina@gmail.com](mailto:lemsaraamina@gmail.com)

[michael.piechotta@uni-heidelberg.de](mailto:michael.piechotta@uni-heidelberg.de)

[felix.gm.ernst@outlook.com](mailto:felix.gm.ernst@outlook.com)

[ludivine.wacheul@ulb.be](mailto:ludivine.wacheul@ulb.be)

[denis.lafontaine@ulb.be](mailto:denis.lafontaine@ulb.be)

[christoph.dieterich@uni-heidelberg.de](mailto:christoph.dieterich@uni-heidelberg.de)

**Supplementary Table 1:** Oligonucleotides for IVT generation and Nanopore sequencing.

| **18S** |  |  |
| --- | --- | --- |
|  | Primers for IVT generation | TAATACGACTCACTATAGtacctggttgatcctgccag (T7-18S fw)  Taatgatccttccgcaggttc (18S rv) |
|  | ONT adapter | GAGGCGAGCGGTCAATTTTCCTAAGAGCAAGAAGAAGCCtaatgatcct (18S v1 oligo B)  GAGGCGAGCGGTCAATTTTCCTAAGAGCAAGAAGAAGCCtaatgatccttccgcaggtt (18S v2 oligo B) |
| **universal** |  |  |
|  | ONT adapter | /5PHOS/GGCTTCTTCTTGCTCTTAGGTAGTAGGTTC (oligo A) |

**Supplementary Table 2:** Oligonucleotides for CRISPR-Cas9 cell line generation and validation.

| **DIMT1L-Y131G** |  |  |
| --- | --- | --- |
|  | Donor DNA | TGAAAACAGATTTGCCATTCTTTGATACTTGTGTGGCAAATTTGCCTGGGCAGGTATGTCCTCACATTTTCAGGAACATCATACTAACTGTTCCTCTGAT (ssDL003) |
|  | Guide RNA | ATGTGAGGACATACCTGATA (crDL049) |
|  | Diagnostic PCR | GCTATGTTCACCACCTGAACTG (LD4372)  GGTTGTTGCTTGTGAACTTGACC (LD4373) |
| **WBSCR22-D82K** |  |  |
|  | Donor DNA | CTGAGTGGAAGTTATCTGTCAGATGAAGGGCACTATTGGGTGGGACTGAAAATCAGCCCTGCCATGCTGGGTAAGTATGTCCTGTCTGGCACCAGGGTGG (ssDL001) |
|  | Guide RNA | GCATGGCAGGGCTGATATCC (crDL026) |
|  | Diagnostic PCR | GAACTCCTTTACCATGTCC (LD4067)  GCAGGAATTAAAGACCCTC (LD4068) |
| **SNORD13 KO** |  |  |
|  | Guide RNAs | AGAATGGATGTATCGCATTA (crDL051)  TAAATCGATCCTTGAAGTTC (crDL052) |
|  | Diagnostic PCR | AATCACAGAATCTCAGTGGG (LD4467)  AAACTAGGCCACCTGTTATC (LD4468) |
|  | Northern blot probe | GCCCACGTCGTAACAAGGTTCAAGGGTGGC (LD2684) |

**Supplementary Table 3**: Sequence of oligonucleotides used for detection of 18S rRNA modifications.

| **Primer extension** |  |  |
| --- | --- | --- |
|  | m${}_{\text{2}}^{\text{6}}\text{A}$ m${}_{\text{2}}^{\text{6}}\text{A}$ and ac^4^C detection on 18S rRNA helix 45 | CGAGCGAGCGAACGAACGGGC (LD2141) |
|  | Primer extension for m^7^G detection on 18S rRNA | GTACAAAGGGCAGGGACTTAATC (LD2120) |
| **Misincorporation Assay** |  |  |
|  | 18S_H45_rev  (used as RT primer) | TAATGATCCTTCCGCAGGTTCACCTAC |
|  | 18S_H45_fwd  (used for Sanger sequencing) | CGTCGCTACTACCGATTGGATGG |

**Supplementary Table 4**: Run time of tools in rRNA benchmark.

| **tool** | **threads** | **rep** | **time_1 [s]** | **time_2 [s]** | **time_3 [s]** | **mean [s]** | **sd** |
| --- | --- | --- | --- | --- | --- | --- | --- |
| **EpiNano DiffErr** | 1 | 1 | 70.66 | 66.17 | 67.18 | 68.01 | 1.92 |
| **JACUSA2 scores** | 1 | 1 | **28.06** | **29.47** | **28.34** | 28.62 | 0.61 |
| **JACUSA2 scores** | 1 | 123 | 49.22 | **44.98** | 45.12 | 46.44 | 1.97 |
| **EpiNano DiffErr** | 8 | 1 | 55.26 | 54.36 | 53.08 | 54.24 | 0.89 |
| **JACUSA2 scores** | 8 | 1 | 88.92 | 88.41 | 88.13 | 88.49 | 0.33 |
| **JACUSA2 scores** | 8 | 123 | **48.72** | 45.33 | **44.15** | 46.06 | 1.94 |
| **Eligos2** | 8 | 1 | 147.28 | 149.52 | 168.51 | 155.10 | 9.52 |
| **Nanocompore** | 8 | 1 | 1306.03 | 1275.48 | 1243.38 | 1274.96 | 25.58 |
| **Nanocompore** | 8 | 123 | 4308.22 | 4230.77 | 4173.13 | 4237.37 | 55.35 |
| **xPore** | 8 | 1 | 653.60 | 621.96 | 616.46 | 630.67 | 16.37 |
| **xPore** | 8 | 123 | 2191.97 | 2171.71 | 2177.88 | 2180.52 | 8.48 |

**Supplementary Figures**


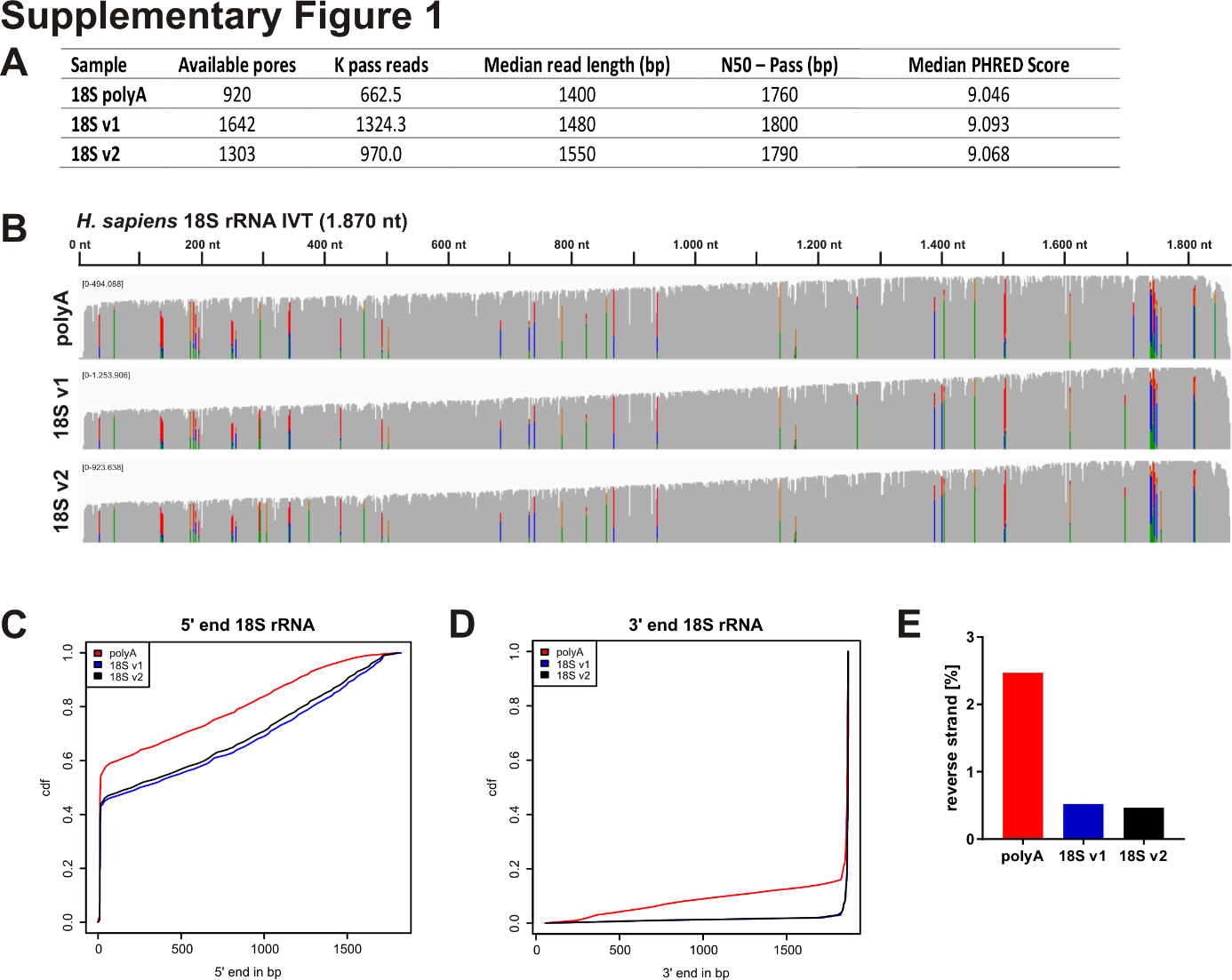


**Supplementary Figure 1:** A) Read statistics of Nanopore direct RNA-seq of unmodified 18S *in vitro* transcripts (IVT) with the standard oligo(dT) adapter (after polyadenylation of the IVT), 18S v1 (20-nts long) and 18S v2 (10-nts long) specific adapters on MinION R9.4.1 flow cells. B) Coverage of 18S IVT from IGV snapshots of the sequencing runs listed in A). Allele frequency threshold = 0.2. C) Cumulative distributive function (cdf) of the 5′ read ends of the 18S rRNA IVT sequenced with either of the three adapters, as indicated. D) Cumulative distributive function (cdf) of the 3′ read ends of the 18S rRNA IVT sequenced with either of the three adapters, as indicated. E) Proportion of reads mapping to the reverse strand for 18S IVT sequencing runs.


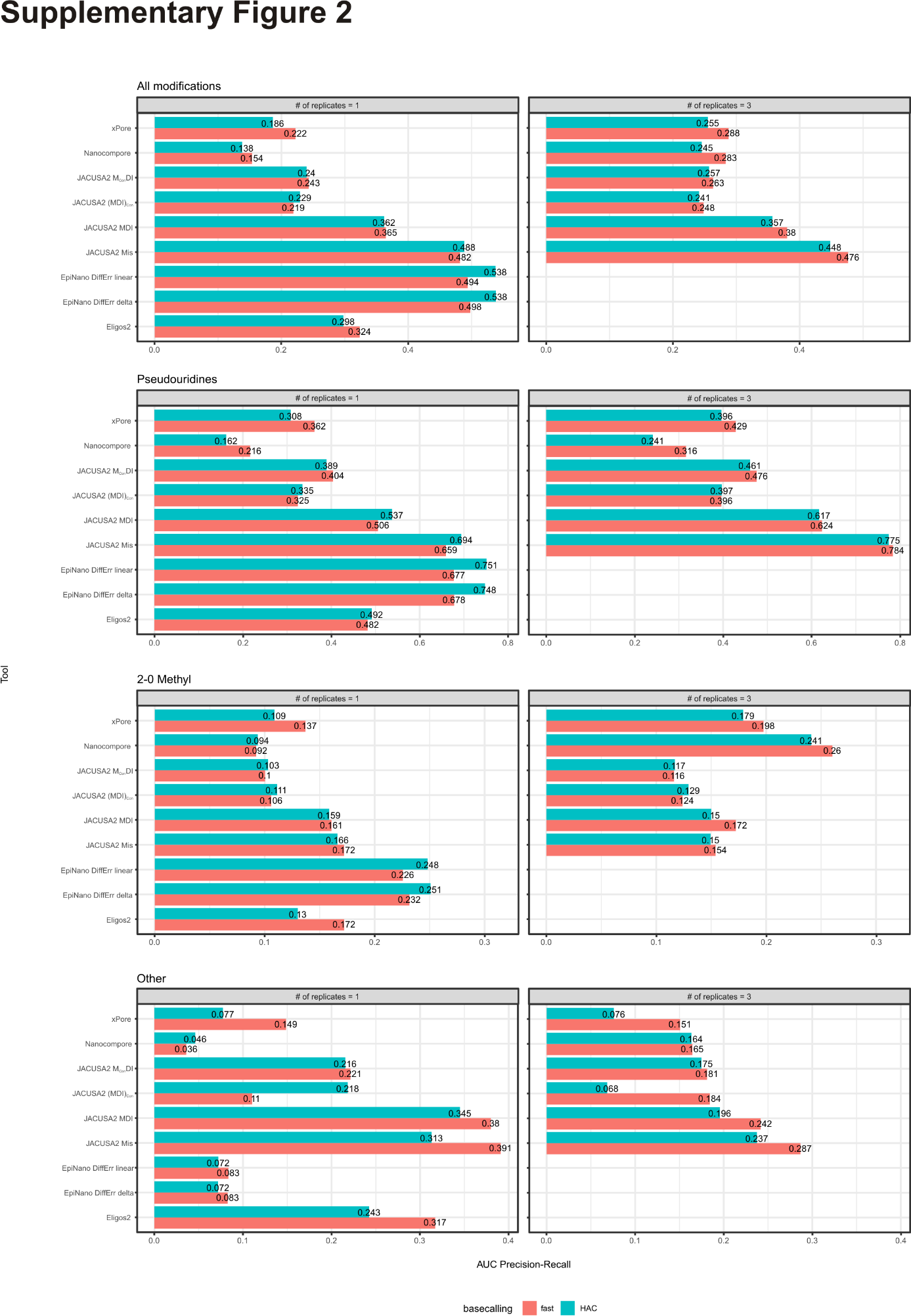


**Supplementary Figure 2.** Benchmark of tools designed for detection of RNA modifications on human 18S rRNA derived from HCT116 cells compared to an 18S IVT. All data sets were downsampled to 5000 reads per sample and basecalled using either the fast (red) or high accuracy (HAC, blue) basecalling mode. Left panels: analysis of a single replicate, right panels: analysis of three replicates. For EpiNano and Eligos2 a replicate analysis is not possible. Panel 1: AUC of a precision-recall analysis for all modifications. Panel 2: AUC of a precision-recall analysis for pseudouridine sites. Panel 3: AUC of a precision-recall analysis for 2’-O-methylation of ribose (Nm) sites. Panel 4: AUC of a precision-recall analysis for all other modifications found on the 18S rRNA (ac^4^C, m^1^acp^3^psU, m^6^A, m${}_{\text{2}}^{\text{6}}\text{A}$ and m^7^G).


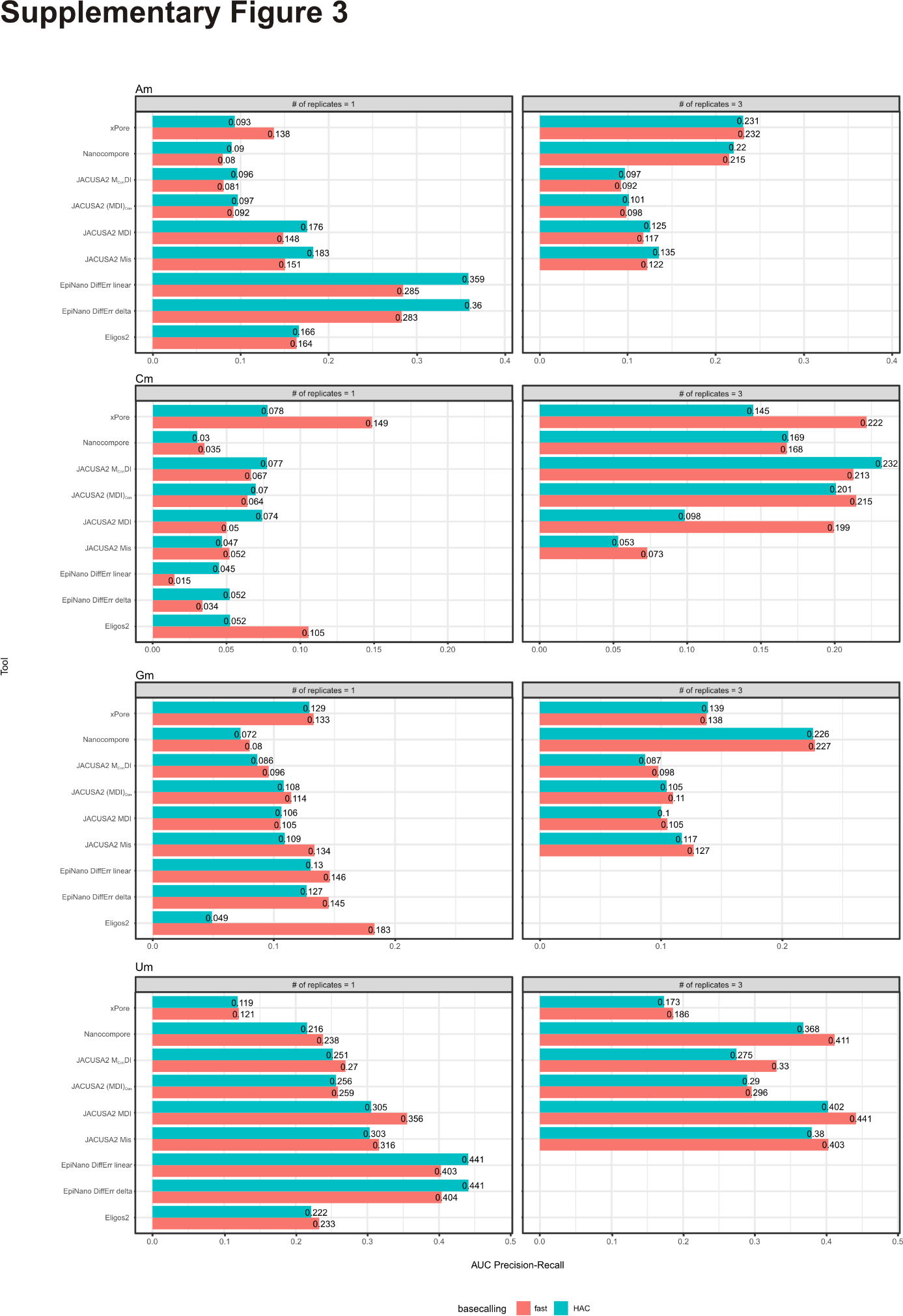


**Supplementary Figure 3.** Stratification of 2’-O-ribose methylation sites. Panel 1: AUC of a precision-recall analysis for Am modifications. Panel 2: AUC of a precision-recall analysis for Cm modifications. Panel 3: AUC of a precision-recall analysis for Gm modifications. Panel 4: AUC of a precision-recall analysis for Um modifications.


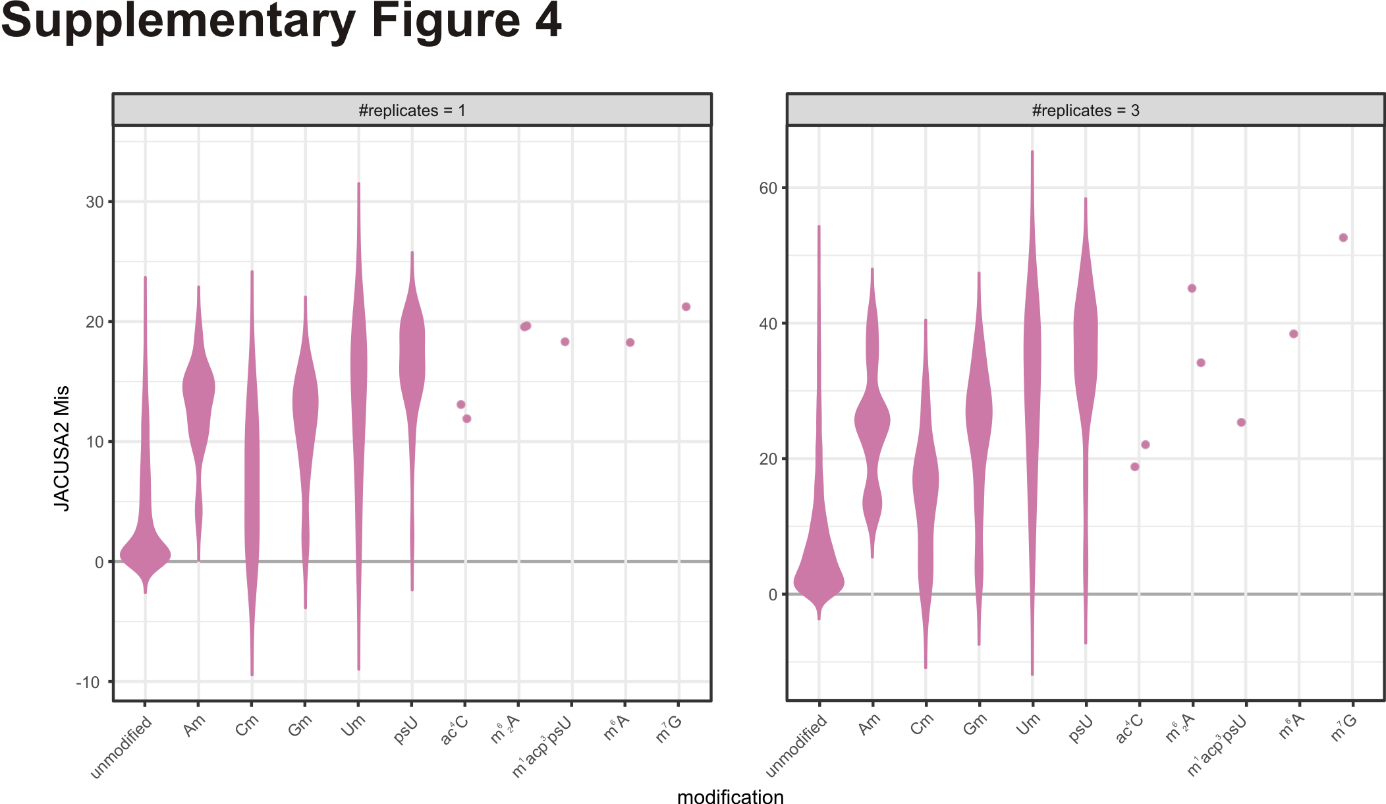


**Supplementary Figure 4.** Violin plot summarizing the JACUSA2 call-2 analysis of the 18S rRNA from HCT116 WT cells and 18S IVT. Shown is the JACUSA Mis score for all modification types on HAC basecalled data as indicated. Left panel: analysis of a single replicate, right panel: analysis of three replicates.


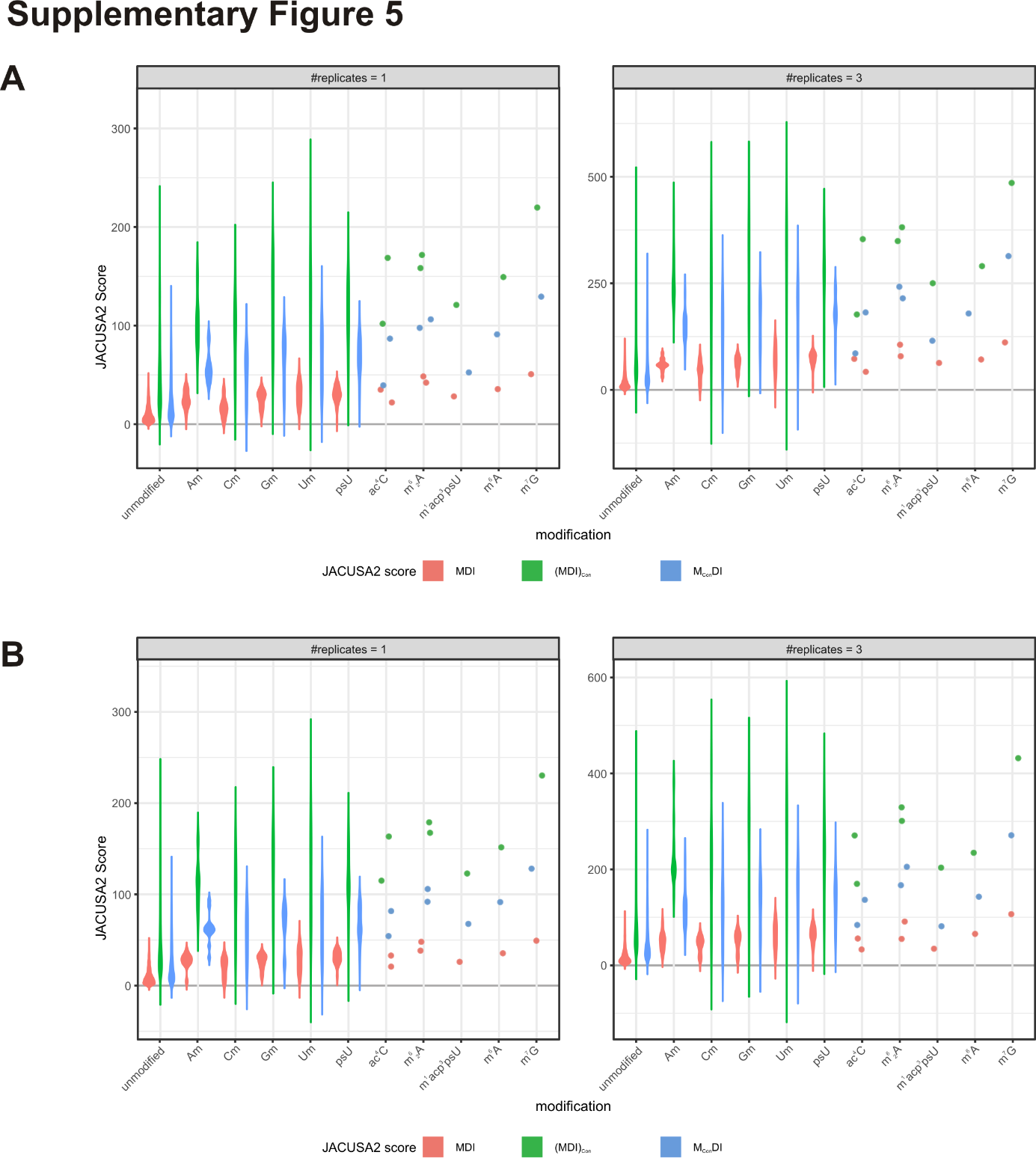


**Supplementary Figure 5.** A) Violin plot summarizing the JACUSA2 call-2 analysis of the 18S rRNA from HCT116 WT cells and 18S IVT. Shown are the JACUSA MDI, (MDI)_Con_ and M_Con_DI scores for all modification types on fast basecalled data as indicated. Left panel: analysis of a single replicate, right panel: analysis of three replicates. B) Violin plots as in A on HAC basecalled data.


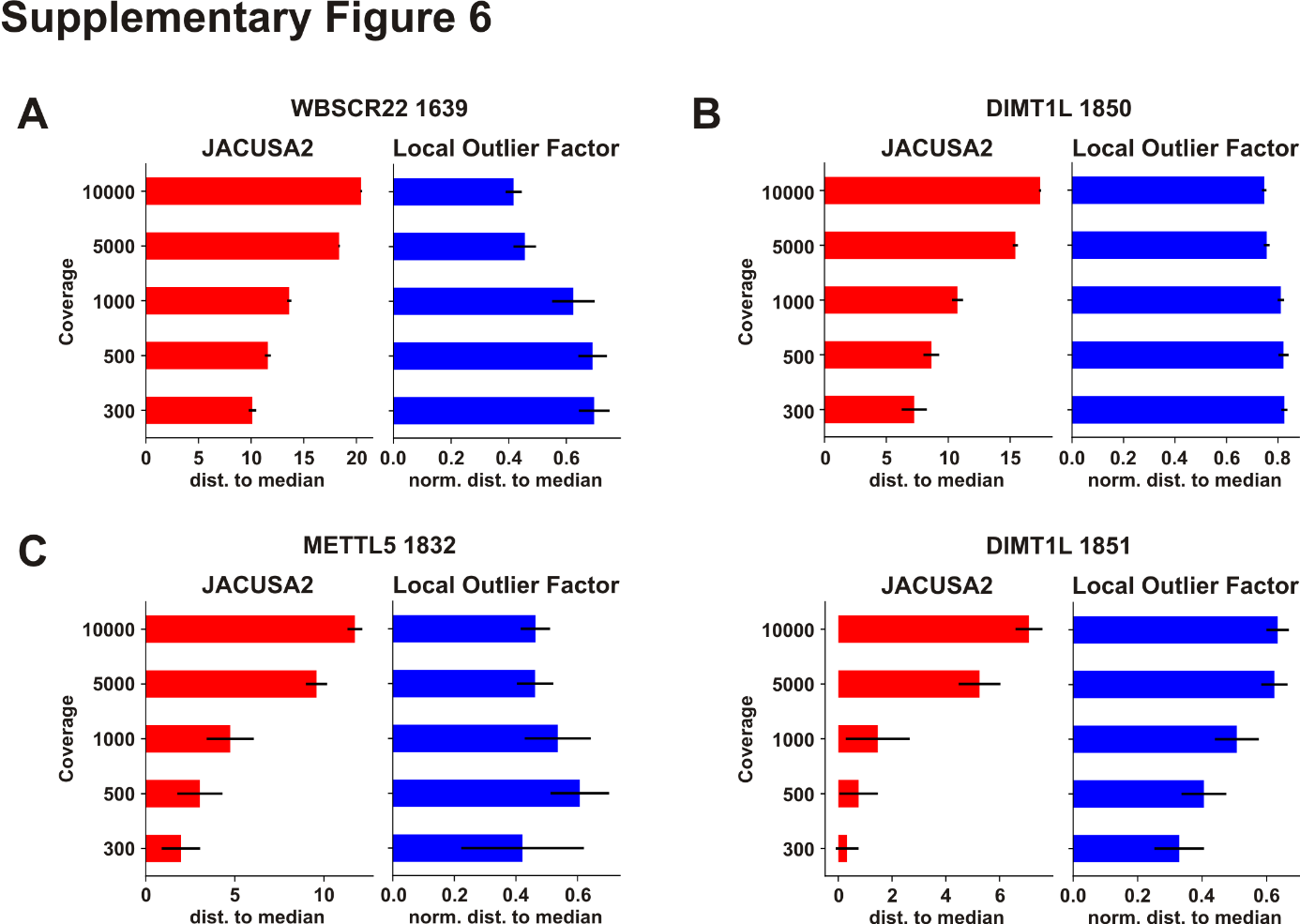


**Supplementary Figure 6.** Detection of rRNA modifications with different read numbers. Indicated read numbers were sampled from MinION sequencing data and analyzed by JACUSA2 in pairwise comparisons. A) Downsampling analysis of WBSCR22-catalyzed m^7^G_1639_. B) Downsampling analysis of METTL5-catalyzed m^6^A_1832_. C) Downsampling analysis of DIMT1L catalyzed m${}_{\text{2}}^{\text{6}}\text{A}$_1850_. D) Downsampling analysis of DIMT1L catalyzed m${}_{\text{2}}^{\text{6}}\text{A}$_1851_. Left panels: Distance of the JACUSA2 Mis score for the respective target site to the median JACUSA2 score for the WT versus KO/MUT comparison. Right panels: normalized distance of the LOF score for the target site to the median LOF score. Shown are the mean and the standard deviation from down sampling employing different seeds (n = 15).


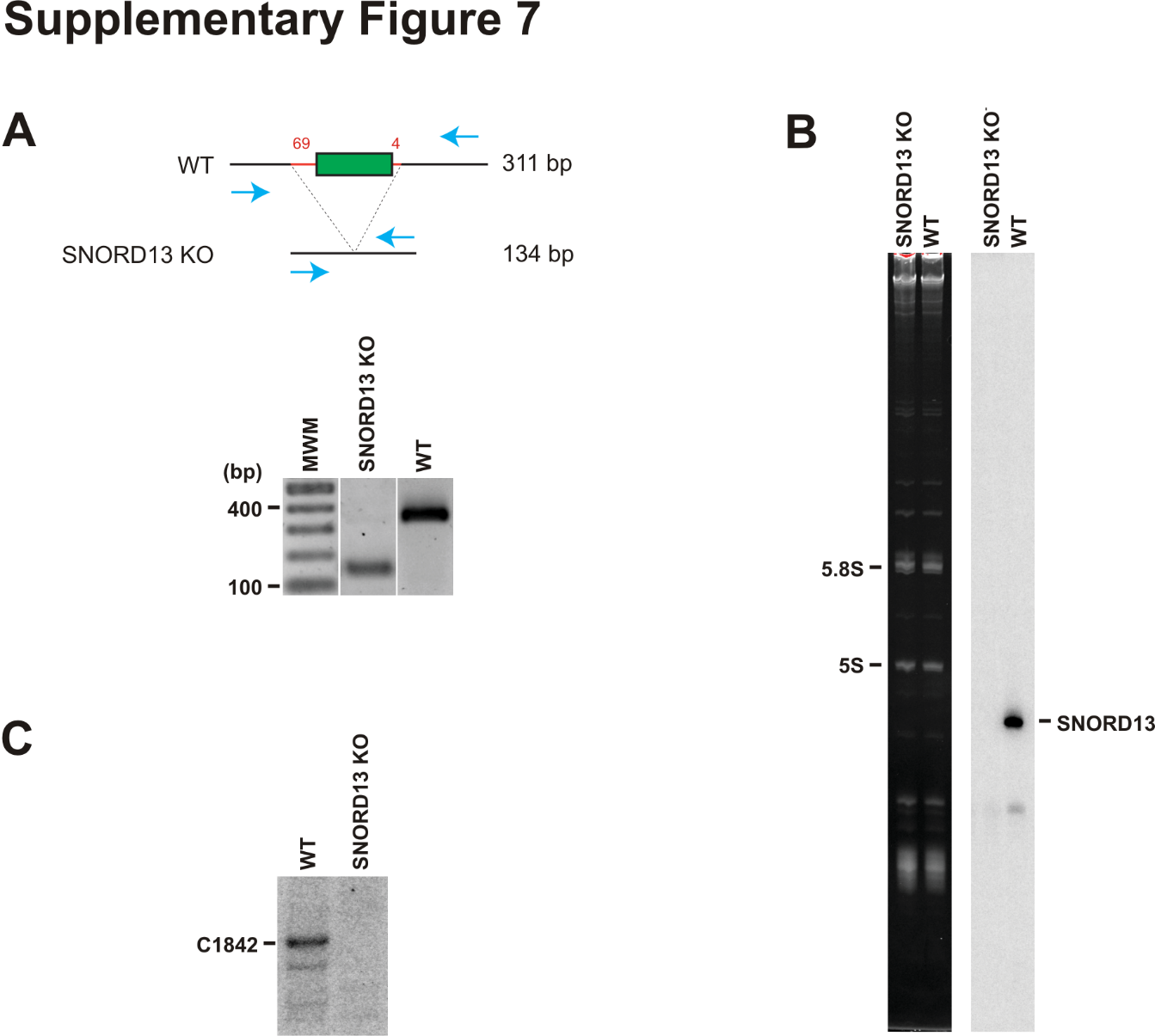


**Supplementary Figure 7:** Generation of the HCT116 SNORD13 KO cell line. A) The gene encoding the snoRNA U13 (SNORD13) was removed from both alleles of HCT116 cells by use of two CRISPR-Cas9 RNP complexes, one on each side of the gene. The entire sequence encoding SNORD13 was removed with an additional 69 nts upstream and 4 nts downstream. The deletion was diagnosed by differential PCR, and by DNA sequencing of the targeted genomic region (not shown). B) The loss of SNORD13 was demonstrated by northern blotting (probe: LD2684). Left panel, ethidium-bromide staining used to control loading. C) Loss of ac^4^C_1842_ on helix 45 of the 18S rRNA was assessed by primer extension following NaBH_4_ treatment, as described with oligonucleotide LD2141.

**Supplementary text: rRNA Benchmark**

**Data and Processing**

FAST5 files for HCT116 WT1-3 and IVT1-3 runs have been base called with Guppy 5.0.11 with fast and high accuracy mode. Subsequently, FASTQ files have been mapped with minimap2 (v2.22) to the 18S and 28S reference sequences.

Mapped BAM files have been filtered with “samtools -q 10 -F 2323” and only reads mapping to 18S rRNA have been retained for further evaluation.

To account for read coverage differences between runs, mapped BAM files have been converted with samtools to FASTQ file format and 5000 reads have been sampled with seqtk (v1.3-r106) by executing: “seqtk sample all_reads.fastq 5000 > 5000reads.fastq”. Finally, reads have been mapped as mentioned above.

**Tools**

The following tools have been considered for comparison:

*Eligos2*

Eligos v2.1.0 has been retrieved from the repository https://gitlab.com/piroonj/eligos2 and a docker container has been built and used with singularity as described in the documentation.

We used “eligos2 pair_diff_mod” to call modification sites:

eligos2 pair_diff_mod \

-tbam $WT_BAM -cbam $KO_BAM \

-reg $BED -ref $REF -t $THREADS -o results \

--max_depth 2000000 --min_depty 5 –esb 0 –oddR 1 –-pval 1

As suggested, when the odds-ratio is < 1, the adjusted p-value has been set to 1. A score has been created from the adjusted p-value by utilizing -log10 values.

*EpiNano DiffErr*

 Epinano v1.2.1 has been installed from the repository https://github.com/novoalab/EpiNano. Epinano DiffErr does not support replicates but offers two modes (linear and delta) to identify RNA modification between a wild type and a control condition.

We followed the procedure documented in the examples in the repository to preprocess BAM files when using the sum of errors feature.

Site weres detected with:

 Rscript --vanilla $EPINANO/Epinano_DiffErr.R \

--coverage 5 \

--wt_sample $WT_EPINANO --ko_sample $KO_EPINANO\

--out_prefix $OUT_PREFIX

--feature sum_err

DiffErr results have been transformed to scores (1 - p-value) where greater values indicate the presence of a modification.

*Nanocompore*

nanocompore v1.0.4 has been installed in a conda environment.

Data preprocessing has been carried out following the instructions in https://nanocompore.rna.rocks/data_preparation.

The following command was used to identify sites:

nanocompore sampcomp \

--file_list1 $WT_COLLAPSE --file_list2 $KO_COLLAPSE \

--label1 wt --label2 ko \

--fasta $REF \

--outpath $OUTPUT_DIR/nanocompore \

--min_coverage 5 \

--allow_warnings \

--nthreads $THREADS

The p-value from statistical test results has been converted to a score by -log(p).

*JACUSA2*

JACUSA2 v2.0.1 has been downloaded from the repository https://github.com/dieterich-lab/JACUSA2.

The following command was used to identify sites:

jacusa2 call-2 \

-p $THREADS \

-q 0 -c 5 \

-D -I \

-r $RESULT \

$WT_BAMS $KO_BAMS

A custom R script was used to parse JACUSA2 results and create auxiliary scores: Mis+Del+Ins, MisContext+Del+Ins, and Mis+Del+Ins_Context.

The test score was extracted from the results and no transformations were carried out.

*xPore*

xpore v2.1 has been installed in a conda environment.

A customized $CONFIG with min reads = 5 and max reads 2000000 was used and the following

command was executed to identify modifications:

xpore diffmod --config $CONFIG --n_processes $THREADS

The p-value from statistical test results has been converted to a score by -log(p).

**Evaluation**

All results from the tested tools have been converted to a common BED file format representation. Predictions have been merged and compared with a custom R script against known 18S rRNA modifications. The area under the precision recall curve has been used to compare the performance of different tools.

**Running time comparison**

Running times were captured using the features of snakemake to account for variance in running time, each tool has been run 3 times. Running time has been measured of all necessary preprocessing and actual identification steps. EpiNano and JACUSA2 scores were run on 1 and 8 threads, for all other tools only 8 threads were feasible.
